# The role of inflammation induced by necroptosis in the development of fibrosis and liver cancer in novel knockin mouse models fed a western diet

**DOI:** 10.1007/s11357-024-01418-3

**Published:** 2024-11-08

**Authors:** Ramasamy Selvarani, HoangVan Michelle Nguyen, Natesan Pazhanivel, Muthusamy Raman, Sunho Lee, Roman F. Wolf, Sathyaseelan S. Deepa, Arlan Richardson

**Affiliations:** 1 Biochemistry & Physiology, University of Oklahoma Health Sciences Center, Oklahoma City, OK USA; 2 Nutritional Sciences, University of Oklahoma Health Sciences Center, Oklahoma City, OK USA; 3 Stephenson Cancer Center, Oklahoma City, OK USA; 4 Oklahoma Center for Geroscience & Healthy Brain Aging, University of Oklahoma Health Sciences Center, Oklahoma City, OK USA; 5 Department of Veterinary Pathology, TANUVAS, Chennai City, Tamilnadu India; 6 Saveetha Medical College, Chennai City, Tamilnadu India; 7 Oklahoma Veteran Affairs Medical Center, Oklahoma City, OK USA

**Keywords:** Chronic liver disease, Hepatocellular adenoma, Liver cancer, Receptor-interacting protein kinase (Ripk3), Mixed lineage kinase domain–like protein (Mlkl)

## Abstract

Non-resolving, chronic inflammation (inflammaging) is believed to play an important role in aging and age-related diseases. The goal of this study was to determine if inflammation induced by necroptosis arising from the liver plays a role in chronic liver disease (CLD) and liver cancer in mice fed a western diet (WD). Necroptosis was induced in liver using two knockin (KI) mouse models that overexpress genes involved in necroptosis (Ripk3 or Mlkl) specifically in liver (i.e., *hRipk3-KI* and *hMlkl-KI* mice). These mice and control mice (not overexpressing Ripk3 or Mlkl) were fed a WD (high in fat, sucrose, and cholesterol) starting at 2 months of age for 3, 6, and 12 months. Feeding the WD induced necroptosis in the control mice, which was further elevated in the *hRipk3-KI* and *hMlkl-KI* mice and was associated with a significant increase in inflammation in the livers of the *hRipk3-KI* and *hMlkl-KI* mice compared to control mice fed the WD. Overexpressing Ripk3 or Mlkl significantly increased steatosis and fibrosis compared to control mice fed the WD. Mice fed the WD for 12 months developed liver tumors (hepatocellular adenomas): 28% of the control mice developing tumors compared to 62% of the *hRipk3-KI* and *hMlkl-KI* mice. The *hRipk3-KI* and *hMlkl-KI* mice showed significantly more and larger tumor nodules. Our study provides the first direct evidence that inflammation induced by necroptosis arising from hepatocytes can lead to the progression of hepatic steatosis to fibrosis in obese mice that eventually results in an increased incidence in hepatocellular adenomas.

**Supplementary Information:**

The online version contains supplementary material available at 10.1007/s11357-024-01418-3.

## Introduction

Chronic, low-grade inflammation that occurs with age (inflammaging) has been observed in all mammalian species studied from rodents to humans [1–3]. Inflammaging is believed to play an important role in aging because: (1) it is strongly associated with a variety of age-related diseases [3], (2) interventions that increase lifespan (e.g., dietary restriction [4], dwarfism [5], and rapamycin treatment [6]) reduce inflammation, and (3) conditions that increase inflammation, e.g., obesity [7–9], are associated with accelerated aging.

Our group has shown that necroptosis, a cell death pathway that triggers inflammation, increases with age in the liver [10], adipose tissue [11], and brain [12] of mice. The age-related increase in necroptosis was associated with increased inflammation in these tissues and inhibiting necroptosis with necrostatin-1 s reduced the age-related increase in inflammation in the liver [13]. In addition, caloric restriction and dwarfism reduce both inflammation and necroptosis in adipose tissue [14]. Our research suggest that necroptosis plays a role in inflammaging, and therefore, necroptosis could play a role in aging and age-related diseases.

The goal of this study was to determine the impact of inflammation triggered by necroptosis on an age-related disease. We chose to study chronic liver disease (CLD) and liver cancer because they increase with age [15, 16] and because chronic inflammation is associated with their occurrence [17]. Currently, liver cancer is the fourth leading cause of cancer-related deaths in the world [18], and the incidence of liver cancer is projected to increase more than 50% globally over the next 20 years [19, 20] largely because of the increase in obesity, which is risk factor for liver cancer [21]. It is projected that half the people in the USA will be obese in 2030 [22]. Interestingly, necroptosis has been shown to increase in the livers of obese mice [23].

In this study, we used two novel knockin mouse models we have developed to induce necroptosis specifically in the liver [24]. These two knockin mice overexpress two genes involved in necroptosis (*Ripk3* or *Mlkl*) specifically in liver (hepatocytes). Using these mice, we were able to test directly the role of necroptosis arising from the liver on the progression of CLD and liver cancer induced by obesity. Obesity was induced by feeding mice fed a western diet (WD), which induces obesity and metabolic perturbations in mice that are common in obese humans [25]. Our data show that overexpressing either Ripk3 or Mlkl specifically in the liver induces necroptosis and inflammation in the livers of obese mice that leads to an increase in steatosis, fibrosis, and the incidence of liver tumors. Our study provides the first data to show that inducing necroptosis in a specific tissue can lead to an acceleration of the progression of an age-related disease.

## Materials and methods

### Animals

Knockin (KI) mice were generated in the C57BL/6 J background by ViewSolid Biotech Inc. (Oklahoma City, OK) using the transgenic constructs as we have described previously (Selvarani et al., 2023). Hemizygous *Ripk3*-KI and *Mlkl*-KI female were crossed to male homozygous for albumin-Cre transgene mice on the C57BL/6 J background (Jackson laboratory, Bar Harbor, CA, stock no#003574) to produce mice that express *Ripk3* or *Mlkl* specifically in the liver, which are designated as *hRipk3*-KI or *hMlkl*-KI mice, respectively. The Cre-transgene is under the regulation of the albumin promoter and is expressed in hepatocytes of embryos starting from E14-21 days onwards [26]. Approximately 50% of pups produced by this cross were *hRipk3*-KI or *hMlkl*-KI mice as expected based on Mendelian inheritance. The *Ripk3*-KI or *Mlkl*-KI littermates do not contain the Cre-transgene and, therefore, do not express the transgenes. We have combined mice from Ripk3-KI and Mlkl-KI mice as the control.

The mice were generated and maintained in the Animal Facility at the Oklahoma City Veterans Affairs Health Care System. Male mice were used in these experiments and were group housed (4 to 5 mice/cage) in ventilated cages at 20 ± 2 °C, on a 12-h/12-h dark/light cycle. At 2 months of age, male mice (5–12 mice/group) were fed ad libitum either a WD containing following % kcal—42% fat from lard, 29% carbohydrate from sucrose, 12.9% carbohydrate from corn starch, 15.2% protein, and 0.203% cholesterol (Teklad custom diet, Envigo, TD.07201)—or a standard laboratory rodent chow diet (CD) c containing following % kcal—13.1% fat, 62.2% carbohydrate, and 24.5% protein (5053 Pico Lab, Purina Mills, Richmond, Indianapolis). The mice were fed these diets for 3, 6, and 12 months and euthanized at 5, 8, and 14 months of age*.* Blood and liver tissue were collected from the mice and weighed, and samples were frozen in liquid nitrogen and stored at − 80 °C until analyzed. The livers were examined for the presence of liver cancer by the appearance of visible surface tumor nodules, and the tissue used for the assays was taken from area of the liver without any tumor. All procedures were approved by the Institutional Animal Care and Use committee at the Oklahoma City Veterans Affairs Health Care System IACUC.

### RNA isolation and quantification of mRNA transcripts

Total RNA was extracted using the RNeasy kit (Qiagen, Valencia, CA) from 20 mg of frozen liver tissue. RT-PCR was performed using a high-capacity cDNA reverse transcription kit (Thermofisher Scientific, Waltham, MA), and quantitative real-time PCR was performed with ABI Prism using Power SYBR Green PCR Master Mix (Thermofisher Scientific, Waltham, MA). The primers for RT-PCR analysis used in this study are given in Supplementary Table S1. The transcript levels of genes involved in various processes that were measured in this study are as follows: macrophage markers (F4/80, CD68, CD206), inflammatory cytokines and chemokines (TNFα, IFNα2, CCl2), fibrosis markers (TGFβ and Col3α1), cancer-related genes (Stat3, VEGF-A, Myc). The relative mRNA transcript levels were determined by a series of calculations. First, the delta CT(∆CT) of the target gene was calculated by subtracting the ∆CT value of the reference gene (β-microglobulin). Next, the delta delta CT (∆∆CT) was obtained by subtracting the ∆CT value of the target sample from the average of ∆CT value of the control samples. Finally, to calculate the fold gene expression values, we used the formula involving the exponentiation of 2 to the power of negative ∆∆CT (2^−ΔΔ*Ct*^). The fold change was then determined by comparing the average ∆CT of the animals in the experimental group to the average of ∆CT of the control group. We also used PCR arrays to measure the expression of genes involved in inflammation and liver cancer: RT^2^ Profiler PCR Array for inflammatory cytokines and chemokines (Cat#330231Z-4E) and the RT^2^ Profiler PCR Array for liver cancer genes (Cat#330231Z-4E).

### Western blotting

Approximately 50 mg of frozen liver tissue was homogenized in RIPA lysis buffer (ThermoFisher Scientific, Waltham, MA) containing 2 mM phenylmethylsulfonyl fluoride and protease inhibitor cocktail (GoldBio, St. Louis, MO). The protein concentration was determined using the Bradford assay (Bio Rad, Hercules, CA), and western blotting conducted as we have described previously [10]. Twenty milligrams of protein was applied to SDS-PAGE gels and transferred onto nitrocellulose membranes after electrophoresis. Immunodetection was carried out by blocking, primary antibody incubation, secondary antibody incubation, and washing. Images were taken using a Chemidoc imager (Bio-Rad, Hercules, CA) and quantified using ImageJ software (U.S. National Institutes of Health, Bethesda, MD). The following primary antibodies were used: Ripk3 from Novus biologicals (Centennial, CO), Mlkl from Millipore Sigma (St. Louis, MO), GAPDH and β-tubulin antibodies from Sigma-Aldrich (St. Louis, MO), and HRP-linked anti-rabbit IgG from Cell Signaling Technology (Danvers, MI) were used as a secondary antibody. To quantify the western blots, the intensity of the protein of interest was divided by the corresponding control band intensity (e.g., either β-tubulin or GAPDH). The intensity of each sample’s band was then divided by the average intensity of control group, thereby expressing the data as a fold change in the protein of interest.

Necroptosis was measured by the appearance of MLKL-oligomers in liver detected using western blots under non-reducing conditions as we have previously described [10]. Briefly, liver tissue was homogenized in HEPES buffer (pH 7.4), and protein samples were prepared using 2 × lamelli buffer at 37 °C for 15 min. Forty micrograms of protein was used, and gels were run under non-reducing conditions without SDS in 1 × transfer buffer and on 7.5% poly-acrylamide gels. MLKL-oligomers were detected and quantified on the gels using the antibody to MLKL as described above for oligomers larger than 200 kDa. Liver from a *Sod1*^*−/−*^ mouse (Sod1KO sample) was used as a positive control on all blots, which allowed us to use this as a standard across all three blots. First, the average intensity of the Sod1KO sample was calculated for each blot. The intensity of the MLKL oligomer was divided by the averaged Sod1KO value for each lane. Subsequently, this result was divided by the intensity of the corresponding control band (e.g., GAPDH). Finally, the intensity of each sample’s band was normalized to the average intensity of the control group, thereby expressing the data as a fold change in the protein of interest.

### Immunostaining

Paraffin-embedded liver sections were incubated with primary antibodies against arginase-1 (Thermo Fisher Scientific, Waltham, MA), Ki67 (Abcam, Cambridge, MA), and cleaved caspase-3 (Cell signaling Technology, Danvers, MA) overnight at 4 °C. Diaminobenzidine-based colorimetric method was used for the detection of target proteins in the sections. Nuclei were counter stained with Mayer’s Hematoxylin (Sigma-Aldrich). Images were taken using a Nikon Ti Eclipse microscope (Nikon, Melville, NY) for three random fields per sample. Staining intensity was quantified using Image J software.

### Plasma levels of alanine transaminase, triglycerides, HMGB1, and albumin

Whole blood was collected in EDTA-coated tubes and left undisturbed on ice for 15–30 min. Plasma was obtained by centrifuging at 1000–2000 × *g* for 20 min at 4°C and collecting the supernatant. Plasma levels of alanine transaminase (ALT) were measured using alanine transaminase colorimetric activity assay kit from Cayman Chemical Company (Ann Arbor, MI) following the manufacturer’s instructions and expressed as U/L. Plasma levels of albumin were measured using colorimetric assay kit from novus bio (Centennial, CO) and expressed as ng/mL. The triglyceride content of plasma was measured using triglyceride colorimetric activity assay kit from Cayman Chemical Company (Ann Arbor, MI) following the manufacturer’s instructions. The lipid content in the plasma was expressed as mg per dL. Plasma levels of the DAMP and HMGB1 were measured using a Mouse HMGB1 ELISA Kit (R&D systems) as per the manufacturer’s instructions and expressed as mg/mL.

### TUNEL staining

TUNEL staining was performed with paraffin-embedded liver Sects. 4 µm by using the DeadEnd™ Colorimetric TUNEL System (Promega, Madison, WI) following the manufacturer’s instructions.

### Assays of steatosis and NASH

In addition to measuring the transcripts of macrophage markers (F4/80, CD68, CD206), and inflammatory cytokines (TNFα, IL-1β, IL-6) (described above), we conducted the following assays to measure steatosis (MAFLD) in liver samples:

o *Measurement of triglyceride levels*—the triglyceride content of liver was measured using triglyceride colorimetric activity assay kit from Cayman Chemical Company (Ann Arbor, MI) following the manufacturer’s instructions. The lipid content of the liver was expressed as mg per g of liver tissue.
o *Histological analysis of liver sections—*formalin-fixed liver tissue was embedded in paraffin, and 4-µm sections were generated using a microtome. Hematoxylin and eosin (H&E) staining was performed on the tissue samples using the standard procedure at the Stephenson Cancer Centre Tissue Pathology Core. H&E-stained sections were digitally scanned at 10× and 20× magnifications using Nikon Ti Eclipse microscope (Nikon, Melville, NY).
o *Oil Red O staining for lipid droplets—*4-µm sections from frozen tissue were stained using the standard procedure with Oil Red O at the Stephenson Cancer Centre Tissue Pathology Core. The Oil Red O staining sections were digitally scanned at 10× and 20× magnifications using Nikon Ti Eclipse microscope (Nikon, Melville, NY). Lipid droplets in the liver tissue sections were identified as red colored droplets in three random, non-overlapping fields per liver sample, and the images of red-colored lipid droplets were captured and quantified using ImageJ software. Quantification was performed on three samples in each group using Image J software, and the results were expressed as the average percentage (%) of area showing Oil Red O staining.
o *Histopathology score for steatosis—*H&E staining was performed in double-blinded study and a total 5–12 slides per group were scored for both macro- and micro-vesicular steatosis. The severity of steatosis was graded based on the percentage of the total area affected using the following categories: 0 (<5%), 1 (5–33%), 2 (33–66%), 3 (>66%).
o *Histopathology score for inflammation—*using H&E staining, inflammatory foci were identified as a group of 10–12 mononuclear cells together and counted manually in a blinded fashion. The number of cell clusters/10mm^2^ were quantified by the counting the number of inflammatory foci/clusters per field using a 10× magnification. This assessment was performed in a double-blinded study and a total 5–12 slides per group were scored for inflammatory foci/clusters. Five different fields were counted, and the average was subsequently scored using the following categories: 0 (None), 1 (<2 foci), 2 (2–4 foci), 3 (>4 foci).

### Assays of fibrosis

In addition to measuring the transcripts of fibrosis markers (TGFβ, Col1α1, and Col3α1), we will conduct the following assays to measure fibrosis in liver samples:

o *Hydroxyproline assay—*the hydroxyproline content of liver was measured as previously described [27]. Frozen liver tissue (~250mg) was pulverized using liquid nitrogen and digested in 6-M hydrochloric acid overnight at 110°C. Ten milliliters of the digest was mixed with 150µL of isopropanol, 75µL of solution A (1:4 mix of 7% chloramine T (Sigma-Aldrich, St. Louis, MO) and acetate citrate buffer (containing 57 g sodium acetate anhydrous, 33.4 g citric acid monohydrate, 435mL 1M sodium hydroxide, and 385 mL isopropanol in 1 L of buffer). The mixture was vigorously mixed and incubated at room temperature for 10 min. Solution B (3:13 mix of Ehrlich’s reagent and isopropanol) was added, and the resulting solution incubated at 58 °C for 30 min. The reaction was stopped by placing on ice for 10 min, and the absorbance at 558nm was measured in a Spectra Max M2 spectrophotometer (Molecular Devices, San Jose, CA). The absorbance values were converted into µg using a standard curve and expressed as µg hydroxyproline/g of tissue.
o *Picrosirius red staining—*formalin-fixed liver tissue was embedded in paraffin, and 4-µm sections were generated using a microtome. Picrosirius red staining was conducted using standard protocol at the Imaging Core facility at the Stephenson Cancer Core. Briefly, formalin-fixed sections were deparaffinized and stained with picrosirius red for 1 h. Excess picrosirius red was removed by rinsing in acidified water, and sections were dehydrated with ethanol and cleared with xylene. The images were taken using a Nikon TI Eclipse microscope (Nikon, Melville, NY) for three random fields per sample. Quantification was performed in three samples in each group using Image J software, expressed as the average percentage (%) of area. Using picrosirius red–stained slides at 10× magnification, hepatic fibrosis was identified based on brunt scoring system. This assessment was part of a double-blinded study, and a total 5–12 slides per group were scored based on the presence of pathologic collagen using the following scoring system: 0 (none), 1 (mild perisinusoidal fibrosis), 2 (zone 3 periportal fibrosis), 3 (bridging fibrosis), 4 (cirrhosis).
o *Masson’s trichrome staining—*staining was conducted using standard protocol at the Imaging Core facility at the Stephenson Cancer Core. Four-micrometer sections of formalin-fixed liver tissue embedded in paraffin were deparaffinized and stained with Masson’s trichrome for 1 h. Excess trichrome was removed by rinsing in acidified water, and sections were dehydrated with ethanol and cleared with xylene. The images were taken using a Nikon TI Eclipse microscope (Nikon, Melville, NY) for three random fields per sample. Quantification was performed on three samples in each group using Image J software, and the results were expressed as the average percentage (%) of area showing Masson’s trichome staining.

### Assays of liver *cancer*

In addition to measuring the transcripts of liver cancer markers (Stat3, VEGF-A, Myc), we conducted the following assays to assess the level of liver cancer in the samples:

o *Gross lesions of a liver cancer—*the liver of each mouse is examined for the presence of liver tumors as identified by the appearance of nodules in the liver. The average number of tumor nodules in each liver was determined on the intact liver as well as the size of the nodules using vernier caliber scale and the number of nodules larger than >5mm determined.
o *Identification of liver cancer cell type*—using H&E staining and immunofluorescence staining for arginase-1, tumor lesions were evaluated to determine the following types of cancer: hepatocellular adenoma, hepatocellular carcinoma, cholangioma, and cholangiocarcinoma.

## Results

### The effect of western diet on necroptosis in hRipk3-KI and hMlkl-KI mice

In this study, male *hRipk3*-KI, *hMlkl*-KI, and control (*Ripk3*-KI and *Mlkl*-KI) mice were fed a WD to induce obesity starting at 2 months of age and compared to mice fed a standard laboratory chow diet (CD). The mice were studied at 5, 8, and 14 months of age, i.e., 3, 6, and 12 months after starting the WD. As shown in Supplementary Figure S1, mice fed the WD showed a significant increase in body weight as well as obesity as determined by the weight of epididymal white adipose tissue (eWAT) and liver compared to mice fed the CD. However, there was no significant difference in the body weights or the weights of the eWAT and liver in the *hRipk3*-KI and *hMlkl*-KI mice compared to control, and *hRipk3*-KI or *hMlkl*-KI mice fed either the CD or WD.

**Fig. 1:**
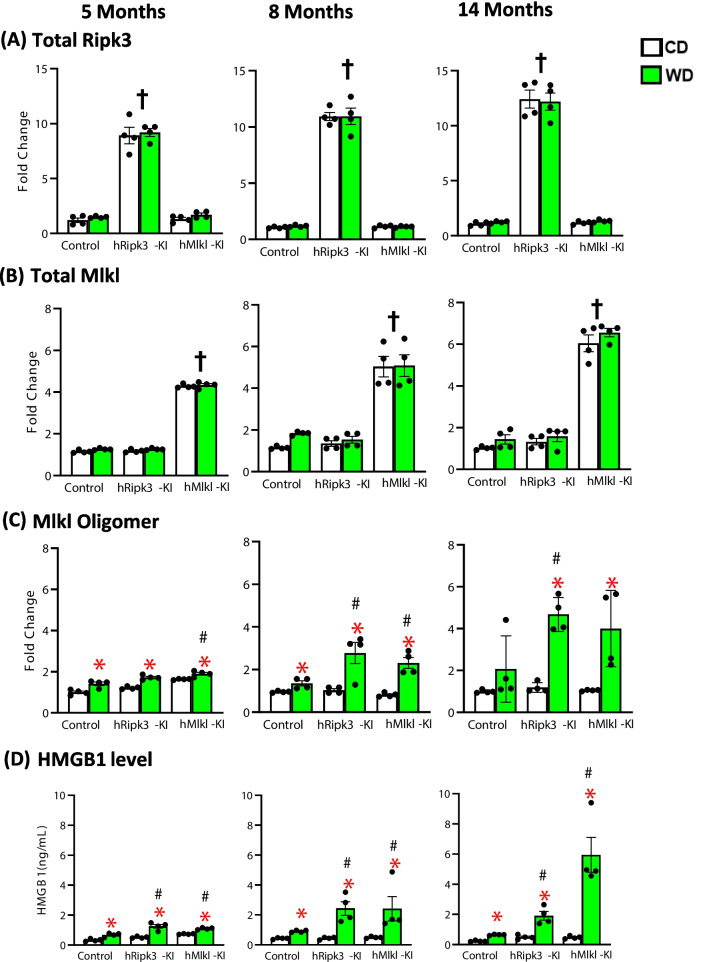
The effect of WD on necroptosis in *hRipk3*-KI and *hMlkl*-KI mice. The levels of Ripk3, Mlkl, and Mlkl-oligomers were measured by western blots (see Supplementary Figure S2 ) in *hRipk3*-KI and *hMlkl*-KI mice and compared to control mice fed either CD (white bars) or WD (green bars). The levels of Ripk3 (**A**) and Mlkl (**B**) normalized to GAPDH are expressed as fold change of Ripk3 or Mlkl expression at the three ages compared to control mice fed CD at 5 months of age. **C** The levels of Mlkl-oligomers (normalized to GAPDH) are expressed as fold change of Mlkl-oligomer expression in liver tissue compared to control mice fed CD at 5 months of age. Liver from a *Sod1*^*−/−*^ mouse (Sod1KO sample) was used as a positive control on all blots, which allowed us to use this as a standard across all three blots. **D** Plasma HMGB1 levels (ng/mL) at 5, 8, and 14 months of age. All data were obtained from four mice/group, expressed as the mean ± SEM, and statistically analyzed using ANOVA. †Significant (*p* ≤ 0.001) difference between mice expressing the *Ripk3* or *hMlkl* transgenes and other mice. *Significant (*p* ≤ 0.05) difference between mice fed CD and WD. #Significant (*p* ≤ 0.05) difference between control mice and *hRipk3*-KI or *hMlkl*-KI mice fed WD

We previously showed that the *hRipk3*-KI and *hMlkl*-KI mice expressed the Ripk3 or Mlkl transgene specifically in liver [24]. Figure 1A, B and Supplementary Figure S2 show that the levels of Ripk3 and Mlkl are ~ 10- and ~ fivefold higher, respectively, in the livers of *hRipk3*-KI and *hMlkl*-KI mice compared to control, and *Ripk3*-KI or *Mlkl*-KI mice at all three ages studied, which is similar to the increased expression of Ripk3 and Mlkl we previously reported in liver for these mice [24]. Importantly, the overexpression of Ripk3 and Mlkl was the same for mice fed either the CD or WD (Fig. 1A, B). Because *Ripk3*-KI and *Mlkl*-KI mice showed no expression of either transgene (Supplementary Figure S2 ) as we have previously reported [24] and because we did not observe any difference in these two KI mouse models in any of the parameters measured below, the control mice consist of equal numbers of *Ripk3*-KI or *Mlkl*-KI mice. To determine the impact of WD on necroptosis in *hRipk3*-KI and *hMlkl*-KI mice, we first measured the levels of Mlkl-oligomers in liver tissue using western blots as shown in Supplementary Figure S2 . Figure 1C shows the level of Mlkl-oligomers in the livers of mice fed a CD or WD at the three ages. At 5 months of age, we observed a modest, but significant increase in Mlkl-oligomers in the mice fed the WD; however, Mlkl-oligomers were only slightly increased in the *hMlkl*-KI fed a WD compared to control mice fed a WD. At 8 and 14 months of age, the level of Mlkl-oligomers dramatically increased in the livers of the *hRipk3*-KI and *hMlkl*-KI mice fed the WD compared to control mice fed WD. We also measured plasma HMGB1 levels as a second measure of necroptosis because HMGB1 is a DAMP, which is released by cells undergoing necroptosis [28]. As shown in Fig. 1D, a significant increase in HMGB1 levels was observed in mice fed the WD compared to mice fed the CD. Importantly, we observed significantly higher plasma levels of HMGB1 at all ages for the *hRipk3*-KI and *hMlkl*-KI mice fed the WD compared to control mice fed the WD, and this difference increased with the time the mice were fed the WD. In Supplementary Figure S3A, we also observed a significant increase in plasma ALT levels (a measure of liver damage/cell death) in the *hRipk3*-KI and *hMlkl*-KI mice fed a WD, which is also consistent with increased necroptosis compared to control mice fed the WD. We also measured plasma albumin levels (Supplementary Figure S3B) in the mice because it is often associated with liver dysfunction, inflammation, and disease [29]. At 8 and 14 months of age, the plasma levels of albumin tended to be reduced in the mice fed the WD with little difference in the *hRipk3*-KI or *hMlkl*-KI fed the WD and the control mice fed WD. However, we observed that apoptosis (caspase-3 activity or TUNEL staining) increased with age in mice fed the WD and was significantly higher in the *hRipk3*-KI and *hMlkl*-KI fed the WD compared to control mice fed the WD (Supplementary Figure S3C, D). Our results are consistent with previous studies showing that feeding a high-fat diet induces apoptosis in liver [30–33]. Because inflammation has been shown to induce apoptosis [34, 35], the increased apoptosis observed in the *hRipk3*-KI and *hMlkl*-KI mice most likely arises from inflammation induced by the increased necroptosis.

### The effect of western diet on inflammation in hRipk3-KI and hMlkl-KI mice

**Fig. 2:**
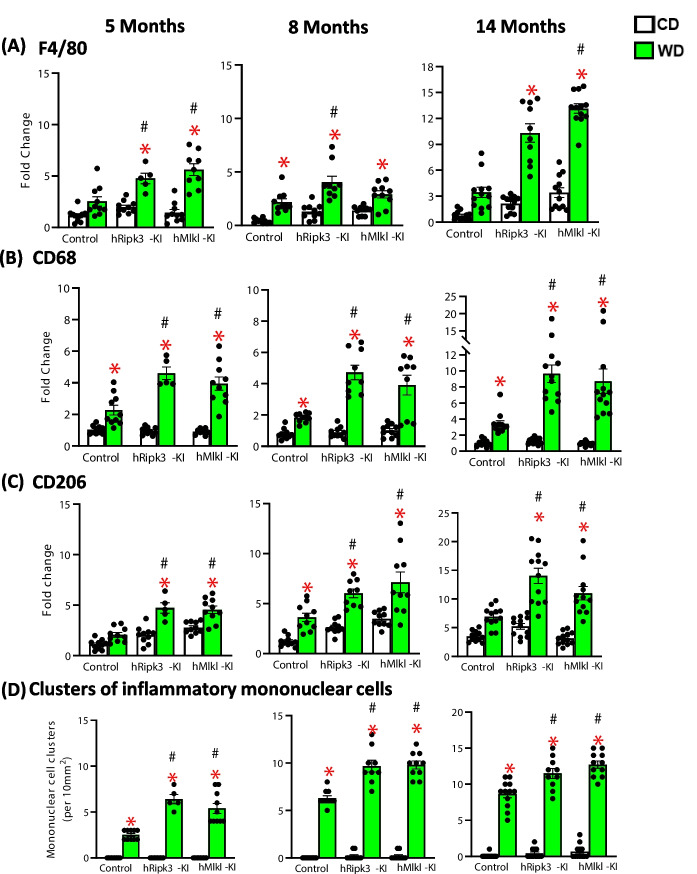
The effect of WD on macrophages in the liver of *hRipk3*-KI and *hMlkl*-KI mice. Markers of macrophages were measured in livers of *hRipk3*-KI or *hMlkl*-KI mice and compared to control mice fed either CD (white bars) or WD (green bars). Transcript levels of the following markers of macrophages were normalized to β-microglobulin and expressed as fold change compared to control mice fed a CD diet at 5 months of age: total macrophages [F4/80, (**A**)], proinflammatory M1 macrophages [CD68, (**B**)], and anti-inflammatory M2 macrophages [CD206, (**C**)]. **D** Quantification of clusters of mononuclear cells from H&E staining (see Supplementary Figure S4) in liver tissue at 5, 8, and 14 months of age. The data were obtained from 5 to 12 mice/group and expressed as the mean ± SEM and were statistically analyzed using ANOVA. *Significance (*p* ≤ 0.05) difference between mice fed CD and WD. #Significance (*p* ≤ 0.05) difference between control mice and *hRipk3*-KI or *hMlkl*-KI mice fed WD

Because necroptosis is associated with increased inflammation [10], we determined if the increase in necroptosis observed in the *hRipk3*-KI and *hMlkl*-KI fed the WD led to increased inflammation. Because the binding of DAMPs released from necroptotic cells activate macrophages to induce an inflammatory response [10], we first measured the markers of macrophages in the liver, i.e., F4/80, CD68, and CD206 mRNA levels, which are markers of total macrophages, M1 macrophages, and M2 macrophages, respectively. As shown in Fig. 2A, B, C, a significant increase in these markers was seen when the *hRipk3*-KI and *hMlkl*-KI mice were fed the WD compared to mice fed the CD. Importantly, the macrophage markers were significantly increased at 8 and 14 months of age for *hRipk3*-KI and *hMlkl*-KI mice fed the WD compared to the control mice fed the WD, except for F4/80 mRNA levels for 14-month-old *hRipk3*-KI mice which was not significant because of the animal-to-animal variation in the samples. We also histologically measured the number of inflammatory foci/clusters mononuclear cells in the livers, and the data in Fig. 2D and Supplementary Figure S4 show an increase in clusters of mononuclear cells in mice fed the WD compared to mice fed the CD. Importantly, we observed at all ages a significantly higher number of clusters of mononuclear cells in the *hRipk3*-KI and *hMlkl*-KI mice fed the WD compared to control mice fed the WD.

**Fig. 3:**
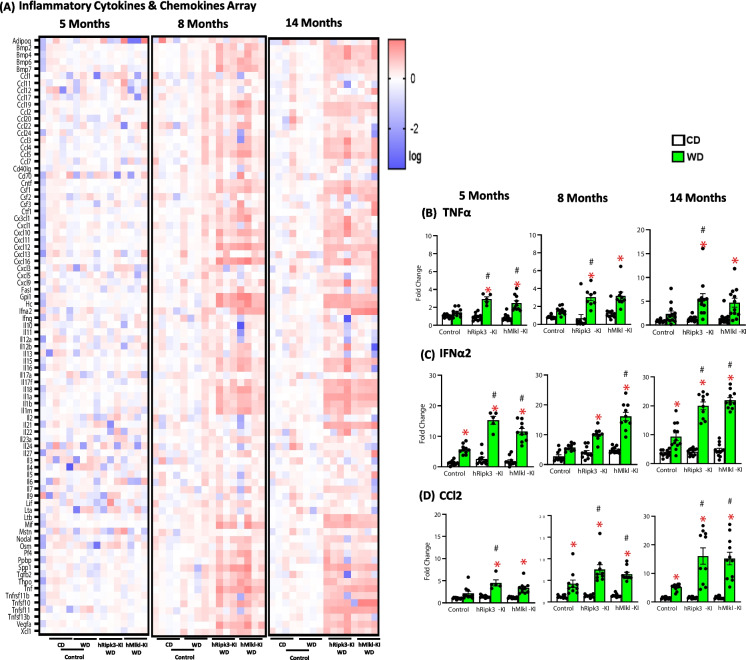
The effect of WD on the expression of chemokines and cytokines in the liver of *hRipk3*-KI and *hMlkl*-KI mice. **A** RT^2^ Profiler PCR Arrays of inflammatory cytokines and chemokines from four animals per group randomly selected from each of the four groups of animals. The intensity of red shows the increasing level of expression of the transcripts. Transcript levels of TNFα (**B**), IFNα2 (**C**), and CCL2 (**D**) from the livers of *hRipk3*-KI or *hMlkl*-KI mice and control mice fed either CD (white bars) or WD (green bars) at 5, 8, and 14 months of age. The data were obtained by rtPCR from 5 to 12 mice/group, normalized to β-microglobulin, and expressed as fold change compared to control mice fed CD at 5 months of age. The data are expressed as the mean ± SEM and were statistically analyzed using ANOVA. *Significance (*p* ≤ 0.05) difference between mice fed CD and WD. #Significance (*p* ≤ 0.05) difference between control mice and *hRipk3*-KI or *hMlkl*-KI mice fed WD

We also measured the transcript levels of proinflammatory cytokines and chemokines in the livers of the mice to assess the effect of overexpressing Ripk3 or Mlkl on the inflammatory status of the liver. Figure 3A shows the heat map of mRNA levels for 96 cytokines and chemokines obtained from four animals randomly selected from each group. At 5 months of age, the panels of cytokines and chemokines were similar for the *hRipk3*-KI and *hMlkl*-KI fed the WD and the control mice fed either the WD or CD. However, at 8 and 14 months of age, the levels of most of the cytokines and chemokines were increased in the livers of the *hRipk3*-KI and *hMlkl*-KI mice fed the WD compared to control mice fed WD. To quantitatively explore in more detail the impact of overexpressing Ripk3 or Mlkl on the expression of specific proinflammatory cytokines and chemokines in the liver of all animals at the three ages, we measured the mRNA levels of two cytokines (TNFα, IFNa2) and one chemokine (CCl2). These cytokines/chemokines play an important role in the regulation of inflammation and their expression appeared to increase in the arrays shown in Fig. 3A. The data in Fig. 3B, C, and D show a significant increase in the transcript levels for three genes which was observed when the *hRipk3*-KI or *hMlkl*-KI mice were fed the WD. In addition, the three transcripts were significantly increased at most ages studied in the livers of the *hRipk3*-KI and *hMlkl*-KI mice fed the WD compared to control mice fed a WD. Thus, the data in Figs. 2 and 3 show that overexpressing Ripk3 or Mlkl had only a slight effect on markers of inflammation in the livers of mice fed the CD. However, when mice were fed the WD, markers of inflammation were significantly increased in the *hRipk3*-KI and *hMlkl*-KI mice and were significantly greater than observed for the control mice fed the WD starting at 5 months of age, i.e., after 3 months on the WD.

### The effect of western diet on chronic liver disease in hRipk3-KI and hMlkl-KI mice

**Fig. 4:**
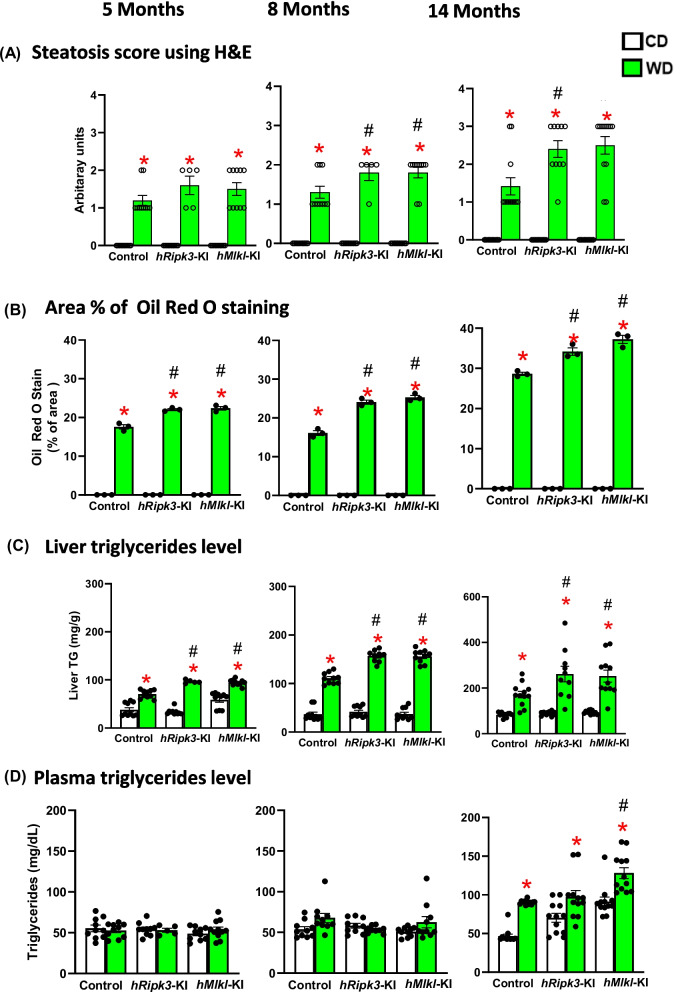
The effect of WD on steatosis in *hRipk3*-KI and *hMlkl*-KI mice. Steatosis was measured in the livers of *hRipk3*-KI and *hMlkl*-KI mice and compared to control mice fed either a CD (white bars) or WD (green bars) at 5, 8, and 14 months of age. **A** Steatosis score from H&E staining (see Supplementary Figure S5A) evaluated for both macro- and micro-vesicular steatosis with the severity of steatosis graded on the percentage of the total area affected using the following categories: 0 (< 5%), 1 (5–33%), 2 (33–66%), 3(> 66%). **B** The percent of area staining for Oil Red O as described in the Materials and Methods. **C** Liver triglycerides levels (mg/g) and **D** plasma triglycerides (mg/dL) were obtained from 5 to 12 mice/group. The data are expressed as the mean ± SEM and were statistically analyzed using ANOVA. *Significance (*p* ≤ 0.05) difference between mice fed CD and WD. #Significance (*p* ≤ 0.05) difference between control mice and *hRipk3*-KI or *hMlkl*-KI mice fed WD

Feeding mice diets high in fat, e.g., WD, leads to fat accumulation in liver (steatosis) and fibrosis, i.e., chronic liver disease (CLD) [36]. Because inflammation plays a major role in CLD [37], we measured steatosis and fibrosis in the livers of the *hRipk3*-KI, *hMlkl*-KI, and control mice fed the WD and CD. First, we used a histology scoring system to measure steatosis severity based on percentage of the area of liver with micro and macro fat droplets (Supplementary Figure S5A). As shown in Fig. 4A, we observed an increase in the steatosis score for the mice fed the WD; however, there was no difference in steatosis score for the *hRipk3*-KI and *hMlkl*-KI fed a WD compared to control mice fed a WD at 5 months of age. At 8 and 14 months of age, the steatosis score was increased in the livers of the *hRipk3*-KI and *hMlkl*-KI mice fed the WD compared to control mice fed WD. The lipid content of the livers was also measured by Oil Red O staining (Fig. 4B and Supplementary Figure S5B) and triglyceride levels. Figure 4B, C shows that a significant increase in Oil Red O staining or the level of liver triglycerides was observed in mice fed the WD compared to mice fed the CD. Again, we observed significantly higher Oil Red O staining or triglyceride levels at all ages in the livers of the *hRipk3*-KI and *hMlkl*-KI mice fed the WD compared to control mice fed the WD, and Oil Red O staining and liver triglyceride levels increased with the time on the WD. Because feeding a high-fat or WD has been shown to increase the triglyceride levels in the plasma [38], we measured plasma triglyceride levels to determine if the clearance of triglycerides by the liver is altered in the *hRipk3*-KI and *hMlkl*-KI mice. As shown in Fig. 4D, there was no significant increase in the level of liver triglycerides in mice fed the WD compared to mice fed the CD until 14 months of age when triglyceride levels in the plasma were increased ~ 50% in the mice fed WD. Only the *hMlkl*-KI mice fed the WD showed higher plasma triglycerides levels compared to control mice fed the WD.

**Fig. 5:**
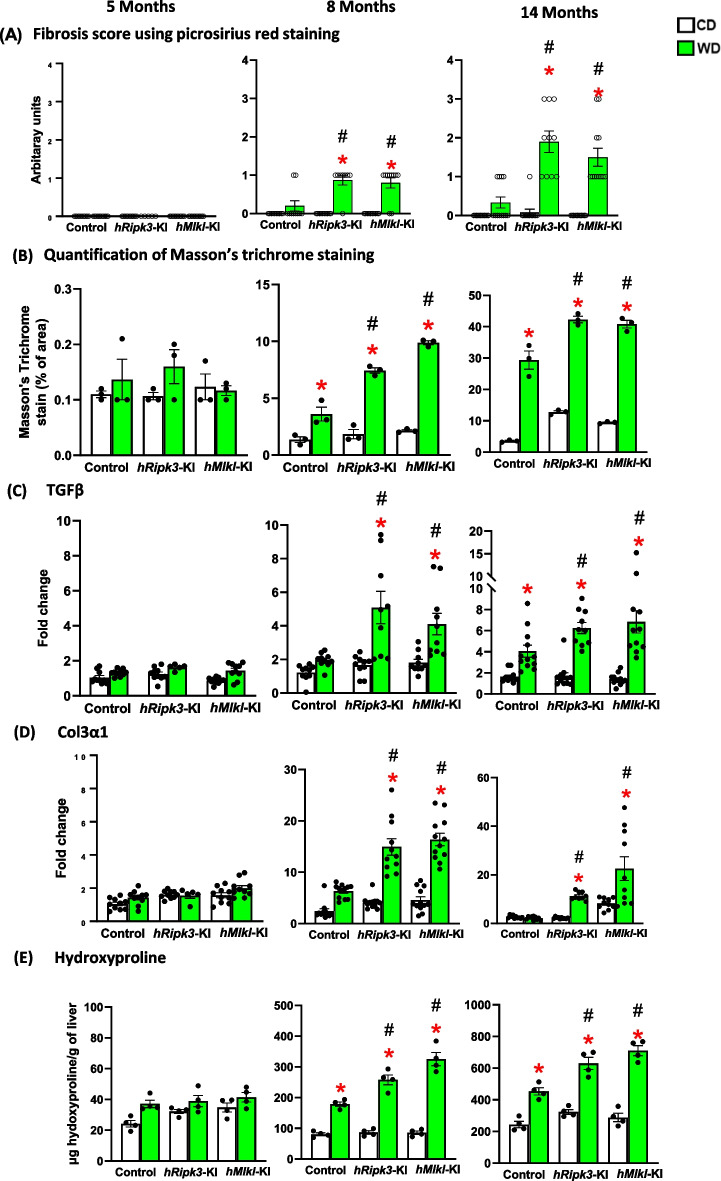
The effect of WD on fibrosis in *hRipk3*-KI and *hMlkl*-KI mice. Fibrosis was measured in the livers of *hRipk3*-KI and *hMlkl*-KI mice and compared to control fed either a CD (white bars) or WD (green bars) at 5, 8, and 14 months of age. **A** Fibrosis score was obtained from Picrosirius red staining (see Supplementary Figure S6A) for 5–12 mice/group and based on the Brunt scoring system for the presence of pathologic collagen: 0 (none), 1 (mild perisinusoidal fibrosis), 2 (zone 3 periportal fibrosis), 3 (bridging fibrosis), 4 (cirrhosis). **B** Quantification from Masson’s trichrome staining was performed for three mice/group (see Supplementary Figure S6B). Transcript levels of TGFβ (**C**) and Col3α1 (**D**) were normalized to β-microglobulin and expressed as fold change compared to control mice fed a CD diet at 5 months of age for 5–12 mice/group. **E** Hydroxyproline levels (µg/g) obtained from four mice/group. The data are expressed as the mean ± SEM and were statistically analyzed using ANOVA. *Significance (*p* ≤ 0.05) difference between mice fed CD and WD. #Significance (*p* ≤ 0.05) difference between control mice and *hRipk3*-KI or *hMlkl*-KI mice fed WD

Next, we used several assays to quantify the severity of fibrosis in the livers of the *hRipk3*-KI, *hMlkl*-KI, and control mice fed the WD. We first measured the fibrosis score using picrosirius red staining, which allows us to detect the collagen fibers resembling chicken-wire configuration with extensive collagen deposition in different regions of the liver as well as bridging fibrosis around the portal triad (Supplementary Figure S6A). Similarly, collagen fiber formation was observed with Masson’s trichrome staining (Supplementary Figure S6B). As shown in Fig. 5A, no fibrosis was detected by picrosirius red staining at 5 months of age; however, fibrosis was detected starting at 8 months of age in the *hRipk3*-KI and *hMlkl*-KI mice fed the WD. At 8 and 14 months of age, there was a significant increase in the fibrosis score for the livers of the *hRipk3*-KI and *hMlkl*-KI mice fed the WD compared to control mice fed WD. Masson’s trichrome staining, which allows us to detect the collagen fibers with extensive collagen deposition in different regions of the liver (Fig. 5B), showed similar results, e.g., increased fibrosis detected starting at 8 months of age and a significant increase in the fibrosis score for the livers of the *hRipk3*-KI and *hMlkl*-KI mice fed the WD compared to control mice fed WD. Next, we measured two markers of fibrosis: transcript levels of TGFβ (Fig. 5C) and Col3α1 (Fig. 5D). TGFβ is a key activator of fibroblasts, promoting a fibrogenic phenotype [39], and Col3α1 is a marker of collagen fiber deposition [40]. We found that the transcript levels for both TGFβ and Col3α1 were the same for the mice fed the WD and CD at 5 months of age. However, at 8 months of age, the transcript levels for both TGFβ and Col3α1were significantly increased for the *hRipk3*-KI and *hMlkl*-KI mice fed the WD, and these fibrosis markers were significantly higher for the *hRipk3*-KI and *hMlkl*-KI mice fed the WD compared to control mice fed the CD. Similar results were observed at 14 months of age. We also measured fibrosis by hydroxyproline content, which is a measure of total collagen in fibrotic tissue [27]. As shown in Fig. 5F, we observed a similar trend in hydroxyproline content of liver as we did for the other measures of fibrosis, and no increase in hydroxyproline content at 5 monhts of age; however, at 8 and 14 months of age, hydroxyproline content was significantly increased in the livers of the *hRipk3*-KI and *hMlkl*-KI mice fed the WD compared to control mice fed WD. Thus, our data show that fibrosis occurs after 6 months of feeding the WD and is consistently higher in the *hRipk3*-KI and *hMlkl*-KI mice compared to control mice fed a WD.

### The effect of western diet on liver *cancer* development in hRipk3-KI and hMlkl-KI mice

**Fig. 6:**
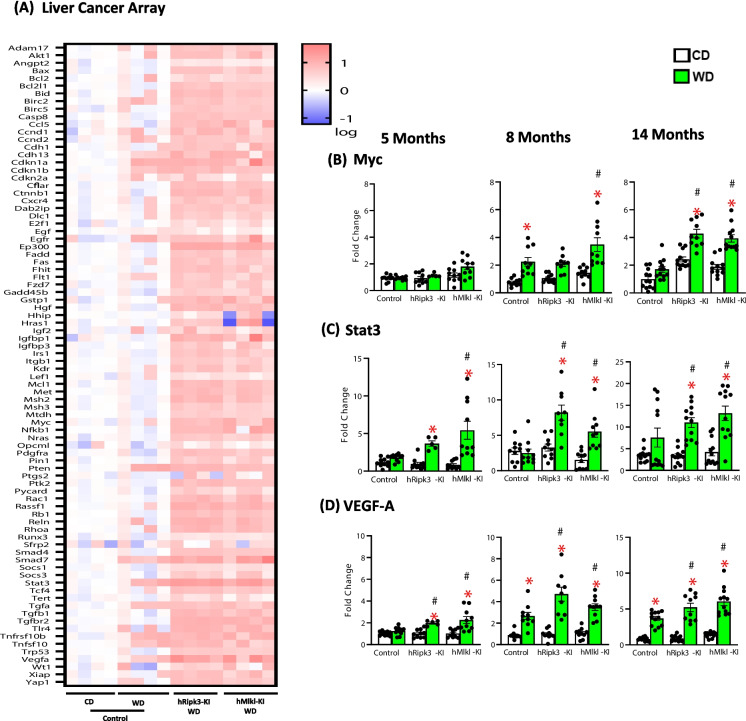
The effect of WD on cancer-related genes in the livers of *hRipk3*-KI and *hMlkl*-KI mice. **A** RT^2^ Profiler PCR Array of cancer-related genes from the livers 14-month-old control mice (*Ripk3*-KI or *Mlkl*-KI mice) fed either CD or WD and *hRipk3*-KI or *hMlkl*-KI mice fed WD. Four mice (two mice with and two mice without tumors) were randomly selected from each group with the intensity of red showing increased level of expression. Transcript levels of Myc (**B**), Stat3 (**C**), and VEGF-A (**D**) were measured by rtPCR at 5, 8, and 14 months of age from livers of *hRipk3*-KI or *hMlkl*-KI mice and control mice fed either CD (white bars) or WD (green bars). Data were obtained from 5 to 12 mice/group, normalized to β-microglobulin, and expressed as fold change compared to control mice fed CD diet at 5 months of age. The data are expressed as the mean ± SEM and were statistically analyzed using ANOVA. *Significance (*p* ≤ 0.05) difference between mice fed CD and WD. #Significance (*p* ≤ 0.05) difference between control mice and *hRipk3*-KI or *hMlkl*-KI mice fed WD

Because MASH is a risk factor for liver cancer [41], we evaluated the impact of overexpressing Ripk3 or Mlkl on liver cancer. First, we measured the expression of genes involved in liver cancer in the *hRipk3*-KI, *hMlkl*-KI, and control mice fed the WD and CD. Figure 6A shows the heat map of the mRNA levels for 96 liver cancer genes obtained at 14 months of age from four animals randomly selected from each group. The levels of most of the liver cancer genes were increased in the livers of the *hRipk3*-KI and *hMlkl*-KI mice fed the WD compared to control mice fed WD. To explore the impact of overexpressing Ripk3 or Mlkl on the potential progression of cancer at 5, 8, and 14 months in the mice, we measured the mRNA levels of three genes, which play a role in the regulation of tumor development and increase in the array (Fig. 6A). The data in Fig. 6B show no increase in Myc mRNA levels at 5 months of age; however, at 8 and 14 months Myc mRNA levels were significantly increased in the livers of the *hRipk3*-KI and *hMlkl*-KI mice fed the WD compared to control mice fed WD. However, Stat3 mRNA levels were significantly increased at 5 months of age in the livers of the *hRipk3*-KI and *hMlkl*-KI mice fed the WD (Fig. 6C), and the Stat3 mRNA levels were higher in the *hRipk3*-KI and *hMlkl*-KI mice fed the WD compared to control mice fed the WD at 8 and 14 months of age. The VEGF-A mRNA levels shown in Fig. 6D are significantly increased by the WD in the *hRipk3*-KI and *hMlkl*-KI mice at all ages and significantly increased compared to mice fed the CD.

We also measured tumor cell proliferation in the livers of the mice by Ki67 immunostaining as shown in Supplementary Figure S7A. The number of Ki67 positive cells per field was significantly increased in mice fed the WD compared to mice fed the CD. We consistently observed significantly higher number of Ki67 positive cells per field at all ages for the *hRipk3*-KI and *hMlkl*-KI mice fed the WD compared to control mice fed the WD, and this difference increased with the time the mice were on the WD.

**Fig. 7:**
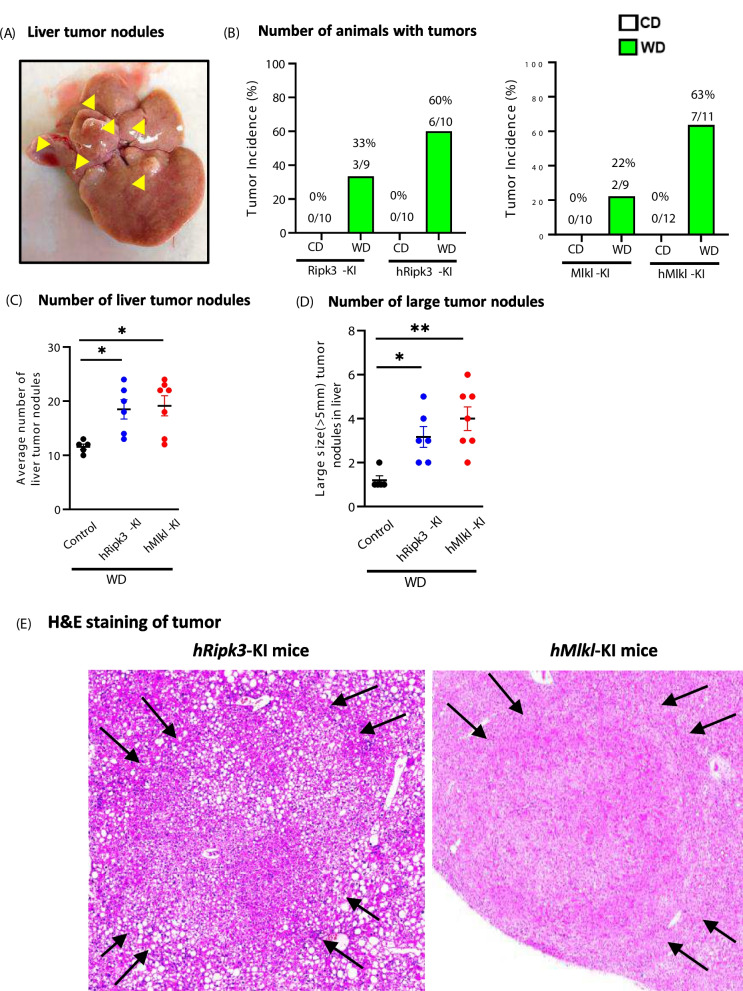
The effect of WD on the incidence of liver tumors in 14-month-old *hRipk3*-KI, *hMlkl*-KI, *Ripk3*-KI, and *Mlkl*-KI mice. **A** Mice with liver tumors were identified by the appearance of tumor nodules in the liver as shown. **B** The number of 14-month-old *hRipk3*-KI, *Ripk3*-KI, *hMlkl*-KI mice, and *Mlkl*-KI mice fed either CD or WD with or without the presence of tumors. To statistically analyze the tumor incidence, the *Ripk3*-KI and *Mlkl*-KI (control) and the *hRipk3*-KI and *Mlkl*-KI mice were combined into two groups. Using a one-tailed chi-square test to analyze these data, we show that the increased incidence liver tumors in the combined *hRipk3*-KI and *hMlkl*-KI mice (62%, 13/21) was significantly higher than in the combined control mice (28%, 5/18) at the *p* = 0.016 level. The average number of tumor nodules (**C**) or average number of large (> 5 mm) tumor nodules (**D**) in the liver of each mouse with a tumor is shown. The data are expressed as the mean ± SEM and were statistically analyzed using ANOVA. *Significance (*p* ≤ 0.05) difference between control mice and *hRipk3*-KI or *hMlkl*-KI mice. **E** H&E staining image from a tumor nodule showing the fibrous encapsulation around the tumor (arrows) by the sharp demarcation of the tumor from the surrounding parenchyma

We conducted a gross pathology examination on each liver for the *hRipk3*-KI, *hMlkl*-KI, *Ripk3*-KI, and *Mlkl*-KI mice fed either the WD or CD at the three ages. We only observed the presence of liver tumors (as shown by the presence of nodules, Fig. 7A) in the 14-month-old mice fed the WD. The tumor incidence in the 14-month-old mice is shown in Fig. 7B, C. No tumors were observed in the mice fed a CD, which is consistent with the literature showing that C57B6 mice do not develop liver cancer when fed a CD [42]. However, 22 to 33% of the control, *Ripk3*-KI, or *Mlkl*-KI mice fed a WD had liver tumors. Importantly, 60 to 63% of the *hRipk3*-KI or *hMlkl*-KI mice fed WD showed the presence of liver tumors. Combining the *Ripk3*-KI or *Mlkl*-KI mice and the *hRipk3*-KI or *hMlkl*-KI mice, we show that 28% (5/18) of the control mice and 62% (13/21) of the *hRipk3*-KI or *hMlkl*-KI mice develop liver tumors which is statistically significant (*p* = 0.0331). We evaluated the progression of tumors in the liver by measuring the number and size of the tumor nodules in each liver. As shown in Fig. 7C, D, the *hRipk3*-KI and *hMlkl*-KI mice with tumors showed a significance increase in the number and size of nodules compared to the control, *Ripk3*-KI, or *Mlkl*-KI mice that had tumors.

Liver tumors can arise from hepatocytes, which give rise to hepatocellular adenoma that progresses to hepatocellular carcinoma and is the major form of liver cancer [43]. However, liver tumors can also arise from cholangiocytes, which generate chlongioma or bile duct adenoma that can progress to intrahepatic cholgiocarcinoma [44, 45]. To identify the type of tumors that were occurring in our mice, a pathologist evaluated multiple tumor nodules from livers of *hRipk3*-KI, *hMlkl*-KI, *Ripk3*-KI, and *Mlkl*-KI mice fed the WD. All liver nodules examined were hepatocellular adenomas as characterized by the tumors being sharply demarcated from the surrounding liver parenchyma with a fibrous encapsulation tissue and hepatocytes with varying size (Fig. 7E).

## Discussion

The general consensus in the research community is that inflammaging (the age-related increase in chronic, low-grade inflammation) plays a role in aging as well as diseases that increase with age, such as cancer, diabetes, and neurodegeneration [46]. Because chronic, low-grade inflammation is associated with obesity [47], the role chronic inflammation plays in aging and age-related diseases will become increasingly important as the rates of obesity increase dramatically in the future in the USA and across the world. Our group has shown that necroptosis, a cell death pathway that triggers inflammation [24], increases with age in various tissues of mice [10, 12] and inhibiting necroptosis reduces inflammation [13]. In addition, necroptosis has been reported to increase with obesity [23, 48, 49]. Because inflammation is one of the hallmarks for both cancer [50] and aging [51], the goal of this research project was to determine if inflammation arising from necroptosis plays a role in obesity-induced CLD and liver cancer. We directly assessed the role of necroptosis in the liver on CLD and liver cancer using two knockin mouse models in which two genes involved in necroptosis (*Ripk3* and *Mlkl*) were overexpressed specifically in liver (hepatocytes): *hRipk3*-KI and *hMlkl*-KI mice [24]. Because Ripk3 and Mlkl are involved in pathways other than necroptosis, it was important for us to show that similar changes in CLD and liver cancer occurred in both *hRipk3*-KI and *hMlkl*-KI mice to demonstrate conclusively that necroptosis was responsible for the changes that we observed. For example, Ripk3 is involved in NLRP3 activation, apoptosis, and lipid metabolism [52, 53], and Mlkl is involved in autophagy and endosomal trafficking [54, 55]. We fed mice a WD high in fat, sucrose, and cholesterol to induce obesity in mice to mimic the metabolic perturbations seen in obese humans [25]. In contrast to choline-deficient diets, which are often used to induce liver cancer and aggressively induce liver tumors in mice within 6 months [56] and do not induce features of the metabolic syndrome seen with obesity [25, 57], WDs require long-term administration before mice develop liver tumors [25]. For example, Van Saun et al. [36] reported that mice develop liver tumors after feeding a high-fat diet (HFD) for 14 months, and Zhang et al. [58] reported that feeding mice a high-fat/high-cholesterol diet for 14 months eventually led to liver tumors. Because liver cancer is an age-related disease that occurs primarily after 60 years of age [59, 60], the WD gives us a more translationally relevant model in which cancer is occurring in older animals when the progression of cancer would normally occur.

We found that feeding mice WD induced necroptosis in the livers of both the control and *hRipk3*-KI and *hMlkl*-KI mice as shown by an increase in both Mlkl-oligomers in the liver and an increase in plasma levels of the DAMP and HMGB1. Wu et al. [61] also found that a WD induced necroptosis in livers of mice. As predicted, we found that overexpressing either Ripk3 or Mlkl increased necroptosis especially after 6 and 12 months on the WD, i.e., at 8 and 14 months of age. To determine if the increased necroptosis in the *hRipk3*-KI and *hMlkl*-KI mice resulted in increased inflammation in the liver, we measured liver macrophages because they are mediators of the inflammatory response in MASH and liver cancer [10]. Markers for total macrophages as well as M1 and M2 macrophages and mononuclear cell clusters were significantly increased in the livers of the *hRipk3*-KI and *hMlkl*-KI mice at 5, 8, and 14 months of age compared to control mice fed WD. Increased hepatic inflammation in the *hRipk3*-KI and *hMlkl*-KI mice was also observed by an increase in the transcript levels for proinflammatory cytokines and chemokines in the liver, e.g., TNFα, which has been implicated in tumor growth and poor prognosis of hepatocellular carcinoma [62], IFNα2, which is increased in obese patients with hepatic steatosis [63] and promotes the recruitment and activation of macrophages [64], and CCl2, which is a strong chemoattractant involved in macrophage recruitment and is a powerful initiator of inflammation [65].

Because chronic inflammation has been proposed to play an important role in CLD [66], we measured hepatic steatosis and fibrosis in the control, *hRipk3*-KI, and *hMlkl*-KI mice fed WD. As expected, steatosis (steatosis score, Oil Red O staining, and triglyceride levels) was increased when mice were fed WD starting at 5 months of age. Steatosis was also significantly increased in the *hRipk3*-KI and *hMlkl*-KI mice fed WD compared to control mice fed WD. Overexpressing Ripk3 or Mlkl in liver also leads to increased fibrosis as measured by the picosirus red staining, Masson’s trichrome staining, markers of fibrosis (transcripts for TGFβ and Col3α1), and hydroxyproline levels. At 8 months of age, we observed a significant increase in all measures of fibrosis in *hRipk3*-KI and *hMlkl*-KI mice fed the WD with little change markers of fibrosis in the control mice fed WD. The severity of fibrosis was increased further at 14 months of age for the mice fed the WD with the *hRipk3*-KI and *hMlkl*-KI mice again showing significantly higher levels of fibrosis than the control mice. Because we found similar increases in the markers for fibrosis in both *hRipk3*-KI and *hMlkl*-KI mice, we have strong evidence that the increase in fibrosis observed in the *hRipk3*-KI and *hMlkl*-KI mice fed the WD was due to increased necroptosis in liver.

Our study is the first to show that inducing necroptosis in the liver can lead to increased hepatic steatosis and fibrosis in mice fed WD. Several studies have shown that blocking/reducing necroptosis globally in all tissues can prevent hepatic steatosis and fibrosis induced by a variety of dietary manipulations. For example, inflammation, steatosis, and fibrosis induced in liver by feeding a choline deficient diet was reduced in Ripk3^−/−^ mice [49, 52]. Majdi et al. showed that treating mice with RIPA-56, an inhibitor of Ripk1, reduced the induction of inflammation, steatosis, and fibrosis in the livers of mice fed a HFD [67]. Using mice genetically engineered to have reduced Ripk1 kinase activity, Tao et al. showed that HFD-induced hepatic steatosis and fibrosis were reduced in the Rip1 kinase-dead knockin mice [68]. While the previous studies have shown that globally reducing necroptosis in all tissues can impact steatosis and fibrosis, our study is the first to show that inflammation arising specifically from necroptosis in the liver of mice plays a role in CLD.

Although a large number of studies have investigated the impact of various high-fat/western diets on hepatic steatosis and fibrosis, there are only two reports on the effect of such diets on liver cancer. VanSaun et al. reported that liver tumor nodules were detected in one of two mice after feeding a HFD (42%) for 9 months, and three of three mice had liver tumors after 14 months [36]. More recently, Zhang et al. reported the appearance of liver tumors in 10% (1 out of 10) of the mice after feeding a high-fat/high-cholesterol diet for 10 months, which increased to 68% (13/19) of the mice with tumors after 14 months of feeding the diet [58]. In our study, 28% (5/18) of the control mice developed liver tumors after feeding a WD (high fat, sucrose, and cholesterol) for 12 months. However, the incidence of liver tumors in the *hRipk3*-KI and *hMlkl*-KI mice was 62% (13/21). Not only was the incidence of liver tumors twice as high in the *hRipk3*-KI and *hMlkl*-KI mice as that in the control mice, but the severity of liver cancer was greater in the *hRipk3*-KI and *hMlkl*-KI mice as shown by an increase in the number and size of nodules in the livers. Interestingly, we did not see any significant increase in the liver mass of the *hRipk3*-KI and *hMlkl*-KI mice compared to the control mice fed the WD as what might have been expected. We believe this is most likely because at 14 months of age the *hRipk3*-KI and *hMlkl*-KI mice are in the early stages of developing liver cancer. With additional time, we would expect these mice to have increased liver mass compared to control mice. Analysis of liver tumors from control, *hRipk3*-KI, and *hMlkl*-KI mice showed that all the tumors were hepatocellular adenomas. Our study also provides information on how necroptosis might impact tumor development. Cell proliferation (Ki67 positive cells) was significantly increased in the livers of the *hRipk3*-KI and *hMlkl*-KI mice compared to control mice fed the WD starting at 5 months of age. Transcript levels of VEGF, which is one of the key regulators of vascular angiogenesis and is key factor in tumor growth and metastatic dissemination [69], were significantly increased in the *hRipk3*-KI and *hMlkl*-KI mice fed a WD starting 5 months of age. The expression of Stat3, which mediates several reciprocal interactions between liver cancer cells and stromal cells that modulate chronic inflammation and tumor formation [70], was significantly increased in the livers of *hRipk3*-KI and *hMlkl*-KI mice starting at 8 months of age. And the transcript levels of Myc, which has been called the “grand orchestrator of cancer growth and immune evasion” [71] and was one of the first oncogenes found amplified in hepatocellular carcinoma [72], were significantly increased in both *hRipk3*-KI and *hMlkl*-KI mice at 14 months of age compared to control mice fed the WD.

The development of MASH (metabolic dysfunction–associated steatohepatitis) in obese individuals is recognized as a risk factor for developing hepatocellular carcinoma in humans [73]. Obesity leads to the accumulation of fat in the liver and hepatic steatosis (metabolic dysfunction-associated steatotic liver disease (MASLD)), which when accompanied by inflammation develops into MASH [74]. MASH can progress to hepatic fibrosis and eventually to liver cancer. Because DAMPs released by cells undergoing necroptosis trigger an inflammatory response, necroptosis-induced inflammation could play an important role in the progression of MASLD to MASH and, therefore, in the development of fibrosis and liver cancer in obese individuals. In humans, Mlkl levels have been shown to be positively correlated with fibrotic markers in liver samples from patients with fibrosis/cirrhosis [75], and Ripk3 levels are correlated with increased inflammation and fibrosis in patients with MASH [52]. Our study provides the first evidence that necroptosis arising from hepatocytes can lead to the sequential progression of hepatic steatosis to fibrosis in obese mice that eventually results in an increased incidence in hepatocellular adenomas. It is interesting to speculate that the increase in CLD in the *hRipk3*-KI and *hMlkl*-KI mice fed the WD might also impact other tissues. Research over the past decade shows that cell non-autonomous mechanisms (e.g., parabiosis, serum transfer, and cell ablation) play important roles in driving degenerative changes of aging [76, 77]. The fact that we observed increased HMGB1 plasma levels in the *hRipk3*-KI and *hMlkl*-KI mice fed WD supports this possibility. For example, DAMPs, such as HMGB1, have been shown to induce cellular senescence in tissues [78].

A “two-hit” model was proposed in 1998 by Day and James [79] to explain the mechanism underlying MASH pathogenesis. The “first-hit” is hepatic steatosis, which is the accumulation of lipid in the cytoplasm of hepatocytes that results in an increase in free fatty acids and de novo lipogenesis. The “second hit” triggers a series of cytotoxic events leading to inflammation and MASH [80]. In 2010, a multiple parallel hit hypothesis was proposed in which lipotoxicty in adipose tissue and alterations in the gut microbiome contribute to MASH [81]. It has been proposed that one of the factors involved in the “second-hit” in liver is oxidative stress/damage, which is a result of mitochondrial dysfunction [82] and increased β-oxidation [83–85] arising from steatosis. The increase in oxidative stress is proposed to initiate an inflammatory cascade leading to MASH. However, mTOR activation has also been proposed to play a role in the “second-hit” [82, 86]. For example, feeding rats [87] or mice [88] HFDs activate mTOR in liver, and mTOR is activated in the livers of genetically obese mice [88, 89]. TNFα could also be involved in the “second-hit” as its production is an early event in MASLD [80]. Lipotoxicity triggers the production of TNFα in hepatocytes [90] and obesity enhances its production in liver [91]. In addition, MALFD patients have been reported to have higher circulating TNFα levels than control patients [92–94]. Interestingly, increased oxidative stress/damage [95, 96], mTOR activation [97, 98], and TNFα levels [99] have been observed to increase with age, and these three pathways have all been shown to induce necroptosis [10]. Based on our data, we propose that necroptosis arising from the liver with age plays an important role in the “second-hit” leading to MASH and liver cancer. Oxidative stress/damage, mTOR activation, and/or TNFα levels induced by steatosis or aging in liver triggers necroptosis in liver cells. DAMPs produced by necroptotic liver cells lead to inflammation resulting in MASH, which can progress to fibrosis and liver cancer.

## Presentation

None

## Supplementary Information

Below is the link to the electronic supplementary material.

Supplementary file1 (DOCX 14 KB)

Supplementary file2 (DOCX 16 KB)

## Data Availability

The data that support the findings of the study are available in the manuscript and supplementary material of this article. Correspondence and requests for information should be addressed to A.R.

## Abbreviations

ALT: Alanine aminotransferase
DAMPs: Damage-associated molecular pattern
H&E: Hematoxylin and eosin
HFD: High-fat diet
KI: Knockin
WD: Western diet
MAFLD: Metabolic dysfunction–associated fatty liver disease
MASH: Metabolic dysfunction–associated steatohepatitis
Ripk3: Receptor-interacting protein kinase 3
Mlkl: Mixed lineage kinase domain like
