# Supplementary material for: The role of inflammation induced by necroptosis in the development of fibrosis and liver cancer in novel knockin mouse models fed a western diet
Source: GeroScience. 2024 Nov 8;47(3):2973–94. doi: 10.1007/s11357-024-01418-3 (PMC12181472; doi:10.1007/s11357-024-01418-3)
Supplement: Supplementary file 1 — Supplementary file1 (DOCX 14 KB) [file 11357_2024_1418_MOESM1_ESM.docx]

**Supplementary Table S1** List of antibodies.

| **Antibodies** | **Company name** |
| --- | --- |
| Ripk3 | Novus biologicals (Centennial, Colorado) |
| Mlkl | Millipore Sigma (St. Louis, Missouri) |
| GAPDH | Sigma-Aldrich (St. Louis, Missouri) |
| β-tubulin | Sigma-Aldrich (St. Louis, Missouri) |
| HRP-linked anti-rabbit IgG | Cell Signaling Technology (Danvers, Michigan) |
| TUNEL staining | DeadEnd^TM^ Colorimetric TUNEL System (Promega, Wisconsin) |
| Ki67 | (Abcam, Cambridge, Massachusetts) |
| cleaved caspase-3 | (Cell signaling Technology, Danvers, Massachusetts) |
| arginase-1 | (Thermo Fisher Scientific, Waltham, Massachusetts) |

**Supplementary Table S2** List of PCR primers used to measure the transcript levels of various genes.

| **Transcript** | **Forward Sequence** | **Reverse Sequence** |
| --- | --- | --- |
| F4/80 | 5'-CCCCAGTGTCCTTACAGAGTG-3' | 5'-GTGCCCAGAGTGGATGTCT-3' |
| CD68 | 5’-CCACAGGCAGCACAGTGGAC-3’ | 5’-TCCACAGCAGAAGCTTTGGCCC-3’ |
| CD206 | 5’-ACTACACACTCATCCATTACAACCAA-3’ | 5'-GGCACCTATCACAATCAGGAGGA-3' |
| TNFα | 5’-CACAGAAAGCATGATCCGCGACGT-3’ | 5’- CGGCAGAGAGGAGGTTGACTTTCT-3’ |
| IFNα2 | 5’-ACAGTCCAGAGAGCCATCAACC-3’ | 5’-TCTCTCCACACTTTGTCTCACAC-3’ |
| CCL2 | 5’-TTAAAAACCTGGATCGGAACCAA-3’ | 5’-GCATTAGCTTCAGATTTACGGGT-3’ |
| TGFβ | 5’-ACCATGCCAACTTCTGTCTGGGAC-3’ | 5’-ACAACTGCTCCACCTTGGGCTTG-3’ |
| Col3α1 | 5’-CTGTAACATGGAAACTGGGGAAA-3’ | 5’- CCATAGCTGAACTGAAAACCACC-3’ |
| Stat3 | 5’-AGGAGTCTAACAACGGCAGCCT-3’ | 5’-GTGGTACACCTCAGTCTCGAAG-3’ |
| VEGF-A | 5’-CTGCTGTAACGATGAAGCCCTG-3’ | 5’-GCTGTAGGAAGCTCATCTTCTCC-3’ |
| Myc | 5’-TCGCCTGTCCTCGAGTCC-3’ | 5’-GGTTTGCCTCTTCTCCACAGAC-3’ |
| HPRT | 5’-CTGGTGAAAAGGACCTCTCG-3’ | 5’-TGAAGTACTCATTATAGTCAAGGGCA-3’ |
| β-microglobulin | 5′-CACTGACCGGCCTGTATGC-3′ | 5′-GGGTGGCGTGAGTATACTTGAAT-3′ |
| β-actin | 5′-ATGGATGACGATATCGCTG-3′ | 5′-GTTGGTAACAATGCCATGTTC-3′ |
