# Supplementary material for: The role of inflammation induced by necroptosis in the development of fibrosis and liver cancer in novel knockin mouse models fed a western diet
Source: GeroScience. 2024 Nov 8;47(3):2973–94. doi: 10.1007/s11357-024-01418-3 (PMC12181472; doi:10.1007/s11357-024-01418-3)
Supplement: Supplementary file 2 — Supplementary file2 (DOCX 16 KB) [file 11357_2024_1418_MOESM2_ESM.docx]

Supplementary information

**Supplementary Figure S1:**  **Characterization of the control, *hRipk3*-KI, and *hMlkl*-KI mice fed WD.** Data are shown for *hRipk3*-KI and *hMlkl*-KI mice compared to control mice (*Ripk3*-KI or *Mlkl*-KI mice) fed either CD (white bars) or WD (green bars). (**A & B**) The body weight, epididymal white adipose tissue (eWAT) weight, and liver weight for control mice (black line) and either *hRipk3*-KI or *hMlkl*-KI mice (red line) are shown for 5-12 mice per group.

**Supplementary Figure S2: The western blots used to measure the levels of Ripk3, Mlkl, and Mlkl-oligomers in liver.** The western blots are shown for *hRipk3*-KI, *hMlkl*-KI, and control (*Ripk3*-KI or *Mlkl*-KI mice) mice fed CD or WD at 5- (**A**), 8- (**B**), and 14- (**C**) months of age. The upper band for the *hRipk3*-KI or *hMlkl*-KI mice represents the level of Ripk3 or Mlkl expressed from the transgenes and the bottom band the level of Ripk3 or Mlkl expressed by the endogenous genes. The red boxed area represents the area of the blots that were used to quantify MLKL-oligomers (larger than 200kDa). The R and M for the control mice represents samples from Ripk3-KI (R) and Mlkl-KI (M) mice. Tissue homogenates from the liver of *Sod1^-/-^* mice (Sod1KO) were used as a positive control for the presence of Mlkl-oligomers. Data were obtained from 4 mice per group.

**Supplementary Figure S3: ALT level, triglycerides level of the control, *hRipk3*-KI, and *hMlkl*-KI mice fed WD.** (**A**) Plasma ALT activity (units/L) at 5-, 8- and 14-months of age are shown for 5-12 mice per group. (**B**) Plasma albumin levels (ng/mL) at 5-, 8- and 14-months of age are shown for 4 mice per group. (**C**) Cleaved caspase-3 positive cells were measured in liver tissue at 5-, 8- and 14-months of age as described in the **Materials & Methods** and are shown for 4 mice per group. (**D**) TUNEL positive cells were determined in liver tissue at 5-, 8- and 14-months of age as described in the **Materials & Methods** and are shown for 3 mice per group [these 3 mice are different from the mice used in (**C**)]. All the data are expressed as the mean ± SEM and were statistically analyzed using ANOVA. *Significance (*p*≤0.05) difference between mice fed CD and WD. #Significance (*p*≤0.05) difference between control mice and *hRipk3*-KI or *hMlkl*-KI mice fed WD.

**Supplementary Figure S4: Examples of the H&E staining images used to measure the levels of clusters of mononuclear cells in liver.** Images are shown for *hRipk3*-KI, *hMlkl*-KI, and control mice fed either CD or WD at 5-, 8-, and 14- months of age. The images were scored as part of a double blinded study, and the red arrows indicate clusters of mononuclear cells.

**Supplementary Figure S5: Examples of images used to measure steatosis in liver.**  (**A**) Examples of the H&E staining images used to measure the severity of steatosis in liver are shown for *hRipk3*-KI, *hMlkl*-KI, and control mice fed either CD or WD at 5-, 8-, and 14- months of age. The images were scored as part of a double blinded study by pathologists evaluating both macro- and micro-vesicular steatosis with the severity of steatosis graded on the percentage of the total area affected. (**B**) Examples of the Oil Red O staining images used to measure the severity of steatosis in liver are shown for *hRipk3*-KI, *hMlkl*-KI, and control mice fed either CD or WD at 5-, 8-, and 14- months of age. The images were quantified based on the percentage of the total area affected.

**Supplementary Figure S6: Examples of images used to measure fibrosis in liver.**  (**A**) Examples of Picrosirius red staining images used to obtain the fibrosis score in liver are shown for *hRipk3*-KI, *hMlkl*-KI, and control mice fed either CD or WD at 5-, 8-, and 14- months of age. The red arrow indicates collagen fibers resembling chicken-wire configuration. The images were scored as part of a double blinded study by pathologists based on the presence of pathologic collagen: mild perisinusoidal fibrosis, zone 3 periportal fibrosis, and bridging fibrosis. (**B**) Examples of Masson’s trichrome staining images used to quantify collagen fiber formation in liver are shown for *hRipk3*-KI, *hMlkl*-KI, and control mice fed either CD or WD at 5-, 8-, and 14- months of age. The blue color indicates collagen fibers formation and images were quantified based on the percentage of the total area affected.

**Supplementary Figure S7: Measure of cell proliferation in liver.** (**A**) Examples of the Ki67 immunostaining images used to measure cell proliferation in liver of *hRipk3*-KI, *hMlkl*-KI, and control mice fed either CD or WD at 5-, 8-, and 14- months of age. The red arrows indicate Ki67 positive cells. The images were scored as part of a double blinded study and were obtained from 4 mice/group. (**B**) Quantification of Ki67 positive cells per high power field for mice fed either CD (white bars) or WD (green bars). The data are expressed as the mean ± SEM and statistically analyzed using ANOVA. *Significance (*p*≤0.05) difference between mice fed CD and WD. ^#^Significance (*p*≤0.05) difference between control mice and *hRipk3*-KI or *hMlkl*-KI mice fed WD.
